# Supplementary material for: Fully Immersive Virtual Reality-Based Cognitive Remediation for Adults with Psychosocial Disabilities: A Systematic Scoping Review of Methods Intervention Gaps and Meta-Analysis of Published Effectiveness Studies
Source: Int J Environ Res Public Health. 2023 Jan 14;20(2):1527. doi: 10.3390/ijerph20021527 (PMC9864668; doi:10.3390/ijerph20021527)
Supplement: Supplementary file 1 [file ijerph-20-01527-s001.zip › ijerph-2123194-supplementary.pdf]

**Table S1.** General characteristics of the included studies.

| 8 | Location | Sample size/ Type of study | Type of sample | Control | Measurement | Fully Immersive VR | Type of intervention | Duration | Drop out | Main results |
|---|---|---|---|---|---|---|---|---|---|---|
| Kim et al., 2021 [76] | South Korea | 31 EG 25 CG; RCT | MCI/Cognitively normal individuals | Cognitive training sessions using VR | CRI; CERAD-NB | YES | VR cognitive training program: train memory, attention and executive functions. Supermarket scenario.. | 50–60 min; 2 times a week for 4 weeks | EG 9/31 (29%); CG 3/25 (12%) | No statistically significant differences between groups after intervention |
| Kang et al., 2021 [77] | South Korea | 25 EG 20 CG; RCT | MCI both EG and CG | Usual therapy such as pharmacotherapy | MMSE; DST; TMT A-B; K-BNT; SVLT; COWAT; SCWT; GDS; AES; PANAS-P/N; QoL-AD; ROCFT; VRSQ | YES | VR cognitive training: train multi domain cognitive (attention, executive function and memory, working memory; mathematical calculations, visuospatial function,verbal memory, visual memory, processing speed and working memory). Multiple games. | 20–30 min; 2 times a week for 4 weeks | EG 2/25 (8%); CG 2/20 (10%) | Statistically significant difference between groups in improving quality of life (QoLAD), attention, memory and executive function (DST; SVLT; TMT:SCWT) |
| Zając-Lamparska et al., 2019 [84] | Poland | 75 EG 75 CG; Pilot Study | Mild dementia/Healthy older adults (age 60–89) | VR-based cognitive training using the GRADYS game | DSST; DS; BDT; CTT; TA (d2); BVRT; AVLT; ROCFT; ACE-III; BNT | YES | VR-based cognitive training: divided in four modules (attention, memory, language, and visuospatial processing). The storyline of each module scenario consists of tasks inspired by daily life. Each module has three difficulty levels. GRADYS game. | 45–60 min; 2 times a week for 4 weeks | ES 48/75 (64%); CG 3/75 (4%) | Statistically significant only for the control group in improving cognitive function |
| Maeng et al. 2020 [78] | South Korea | 31 EG 25 CG; RCT | MCI/Cognitively normal individuals | Virtual reality-based cognitive therapy program | CERED; KQOL-AD; GDS | YES | VR-based cognitive therapy program: train multi domain cognitive (memory, attention and executive function). In each session, items were evenly and randomly selected. There were four levels of difficulty from level 1 (four items to buy) to level 4 (seven items to buy); participants chose the difficulty level according to their performance in each session. Supermarket scenario. | 50–60 min; 2 times a week for 4 weeks | EG 7/31 (22%); CG 2/25 (8%) | Statistically significant difference between groups in improving depressive symptoms (GDS), memory, language and executive function (CERED) |
| Liao et al., 2019 [79] | Taipei (Taiwan) | 21 EG 21 CG; RCT | MCI; both EG and CG | Combined Physical (resistance, aerobic and balance exercises) and Cognitive Training (different tasks in ecological scenarios) | TMT; SCWT | YES | VR based Physical and Cognitive training: Physical tasks: simplified 24-form Yang-style Tai Chi, resistance exercise, aerobic exercise, other functional VR daily activities; Cognitive tasks: IADL based scenarios involving orientation, working memory, attention, planning and task switching | 60 min; 3times per week for 12 weeks | EG 3/21 (14,28%); CG 5/21 (23,81%) | No statistically significant differences between groups after intervention cognitive function like executive function (TMT) |
| Kwan et al., 2021 [83] | Hong Kong (China) | 9 EG | MCI and physical fraility | Non-VR motor-cognitive training program: Physical | MoCA; VRSQ | YES | VR simultaneous motor-cognitive training: Cognitive tasks: 8 tasks in ecological virtual scenarios involving visuospatial, calculation, | 30 min; 2 times a week for 8 weeks | EG 1/9 (11,11%); CG 2/8 | No statistically significant differences between groups after intervention. |

| | | | | | | | | | | |
|---|---|---|---|---|---|---|---|---|---|---|
| | | 8 CG; Pilot Study | | task: cycle on the ergometer; Cognitive training: four cognitive games on a tablet computer | | | memory, reaction time and attention; 2 difficulty levels. Motor training: traveling through the virtual world cycling on the ergometer | | (25%) | Positive change in global cognition in the experimental group (MOCA) |
| Park et al., 2020 [84] | South Korea | 12 EG 12 CG; Pilot Study | amnestic MCI (aMCI), both EG and CG | Waiting list | K-MMSE; SGDS-K; SNSB-D; DST; SCWT; WVF | YES | VR cognitive tasks: 6 cognitive games that involved attention, perceptual space skills, numerical ability, perceptivity, logical ability and memory; different level difficulty (i.e., high, middle or low) | 30 min; 2 times a week for 12 weeks | NO DROP OUT | No statistically significant differences between groups after intervention |
| Thapa et al., 2020 [80] | Busan (South Korea) | 34 EG 34 CG; RCT | MCI; both EG and CG | Educational program on general health care (8 sessions - 30/50min) | MMSE-DS; NCGG-FAT; TMT A & B; DSST | YES | VR program: four series of games to aid different cognitive functions: memory, attention and processing speed. EG was also involved in an educational program on general health care | 100 min; 3 times per week for 8 weeks | EG 1/34 (2,94%); CG 1/34 (2,94%). | Statistically significant differences between groups in improving executive function (TMT; DSST) |
| Liao et al., 2020 [81] | Taipai (Taiwan) | 21 EG 21 CG; RCT | MCI, both EG and CG | Combined physical and cognitive training: -physical exercises included resistance, aerobic and balance task; -cognitive task were trained during the physical exercises | MoCA; EXIT-25; AVLT; IADL | YES | VR-based physical and cognitive training: -physical exercises included simplified 24-form Yang-style tai chi, resistance exercises, aerobic exercises and functionally oriented tasks (Kinect system, developed by Tano and Long-Good); -cognitive training included IADL activities (VIVE system, developed by the HTC company) | 60 min (40 min of VR Cognitive training; 20 min of VR physical training); 3 times a week for 12 weeks | EG 3/21 (14,28%); CG 5/21 (23,8%) | Statistically significant differences between groups in improving daily functioning (IADL) |
| La Paglia et al., 2016 [86] | Palermo (Italy) | 9 EG 6 CG; RCT | Schizophrenic disorders, both EG and CG | Integrated Psychological Therapy (IPT) | MMSE; FAB; TMT A/B/B-A; ToL; WCST | YES | VR attention training: hierarchical sequences of tasks; settled in 3 ecological virtual environment, Park (sustained attention task), Valley (selective attention task), Beach (selective and divided attention task) | 90 min; 1 time a week for 10 weeks | Not specified | No statistically significant differences between groups after intervention. Positive change in global cognition (MMSE) and attention (TMT) in the experimental group |
| Hwang et al., 2017 [83] | Daegu (South Corea) | 12 EG 12 CG; RCT | MCI, both EG and CG | Traditional occupational therapy for memory and balance ability | VST; WCT | Not specified | Not specified | 30 min; 5 times a week for 4 weeks | Not specified | Statistically significant differences between groups in improving memory (VST) |

CRI: Cognitive Reserve Index; CERAD-NB: Consortium to Establish a Registry for Alzheimer's Disease Neuropsychological Battery; MMSE: Mini-Mental State Examination; DS: Digit Span; TMT: Trial Making Test; K-BNT: Korean Boston Naming Test; SVLT: Seoul Verbal Learning Test; COWAT: Controlled Oral Word Association Test; SCWT: Stroop Color and Words Test; GDS: Geriatric Depression Scale; AES: Apathy Evaluation Scale; QoL-AD: Quality of Life Akzheimer Disease; PANAS: Positive and Negative Affect Schedule; ROCFT: Roy-Osterrieth Complex Figure Test; VRSQ: Virtual Reality Sickness Questionnaire; DSST: Digit Symbol Substitution Test; BDT: Block Design Test; CTT: Color Trails Test; TA (d2): Test Of attention; BVRT: Benton visual Retention Test; AVLT: Auditory Verbal Learning Test; ACE-III: Addenbroke's Cognitive Examination; BNT: Boston Naming

Test; WVF: Words Verbal fluency; MOCA: Montreal Cognitive Assessment; NCGG-FAT: National Center for Geriatrics and Gerontology functional assessment tool; EXIT-25: Executive Interview; FAB: Frontal Assessment Battery; TOL: Tower of London; WCST: Winsconsing Card Sorting test; VST: Virtual Supermarket Test; WCT: Word Choice Test.

**Table S2.** Effects of fully immersive virtual reality for cognitive remediation in people with Mild Cognitive Impairment.

| Results of meta-analysis | | | | | | | | | | | | Trim-and-Fill | | | | |
|---|---|---|---|---|---|---|---|---|---|---|---|---|---|---|---|---|
| **Outcome** | **K** | **N** | **Model** | **Hedges'g** | **95% CI** | | **z** | ***p*** | **Q** | ***p*** | **I²** | **k added** | **New estimate** | **95% CI** | | ***p*** |
| Executive functions | 6 | 252 | FE | −0.13 | −0.34 | 0.07 | −1.30 | 0.19 | 7.73 | 0.17 | 35% | | | | | |
| | | | RE | −0.15 | −0.49 | 0.18 | −1.19 | 0.29 | | | | 1 | −0.11 | −0.37 | 0.14 | 0.40 |
| Attention | 6 | 242 | FE | −0.15 | −0.34 | 0.04 | −1.53 | 0.12 | 2.33 | 0.80 | 0% | | | | | |
| | | | RE | −0.15 | −0.32 | 0.02 | −2.24 | 0.07 | | | | 0 | | | | |
| Memory | 5 | 176 | FE | 0.45 | 0.21 | 0.69 | 3.71 | 0.0002 | 2.58 | 0.63 | 0% | | | | | |
| | | | RE | 0.45 | 0.18 | 0.73 | 4.63 | 0.001 | | | | 0 | | | | |
| Language | 4 | 152 | FE | 0.46 | 0.20 | 0.71 | 3.52 | 0.0004 | 5.80 | 0.12 | 48% | | | | | |
| | | | RE | 0.48 | −0.13 | 1.09 | 2.51 | 0.087 | | | | 0 | | | | |
| Global cognition | 5 | 183 | FE | 0.36 | 0.14 | 0.57 | 3.28 | 0.0011 | 11.02 | 0.03 | 64% | | | | | |
| | | | RE | 0.41 | −0.11 | 0.94 | 2.17 | 0.096 | | | | 1 | 0.41 | −0.11 | 0.94 | 0.10 |

FE = Fixed-effects model; RE = Random-effects model.

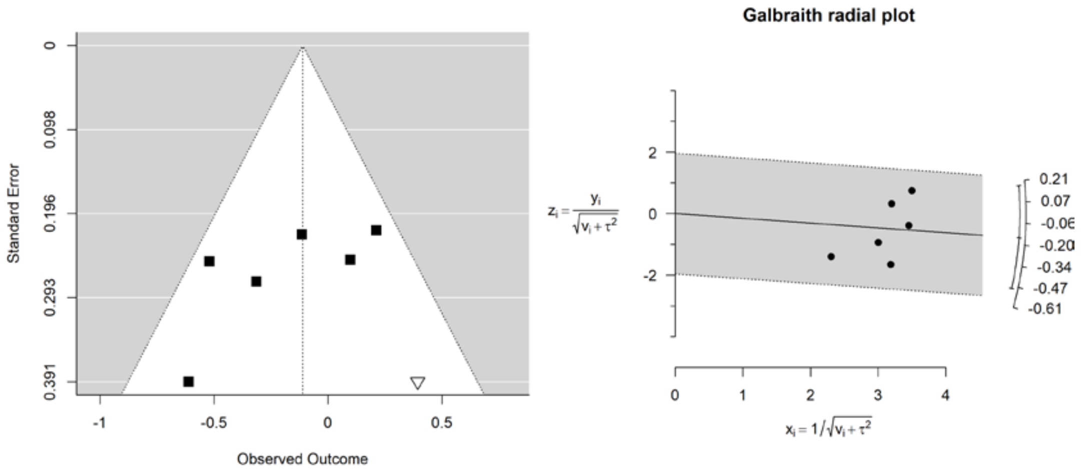


**Figure S1.** EX.

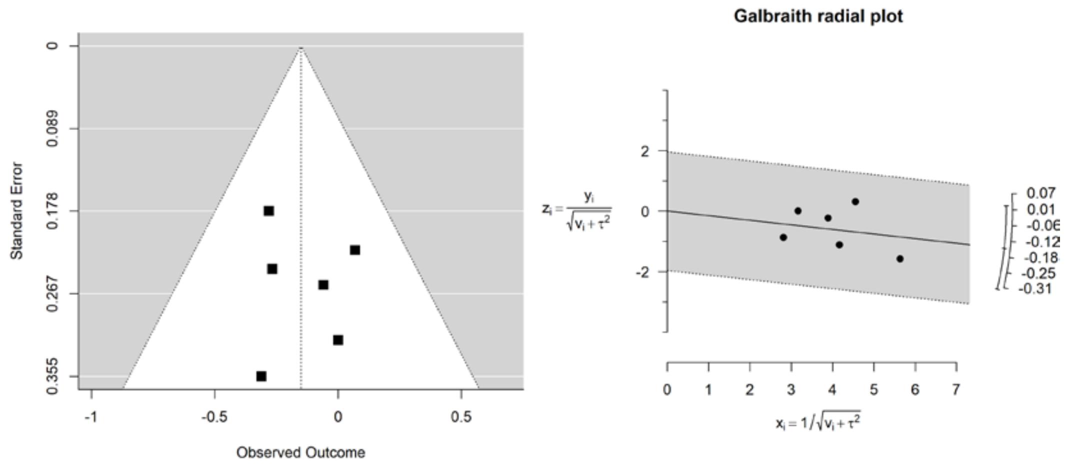


**Figure S2.** ATT.

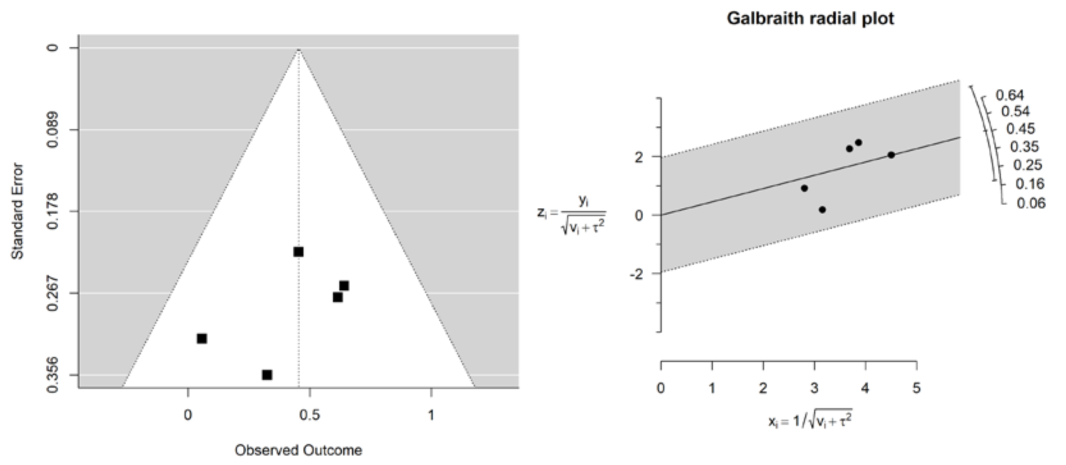


**Figure S3.** MEM.

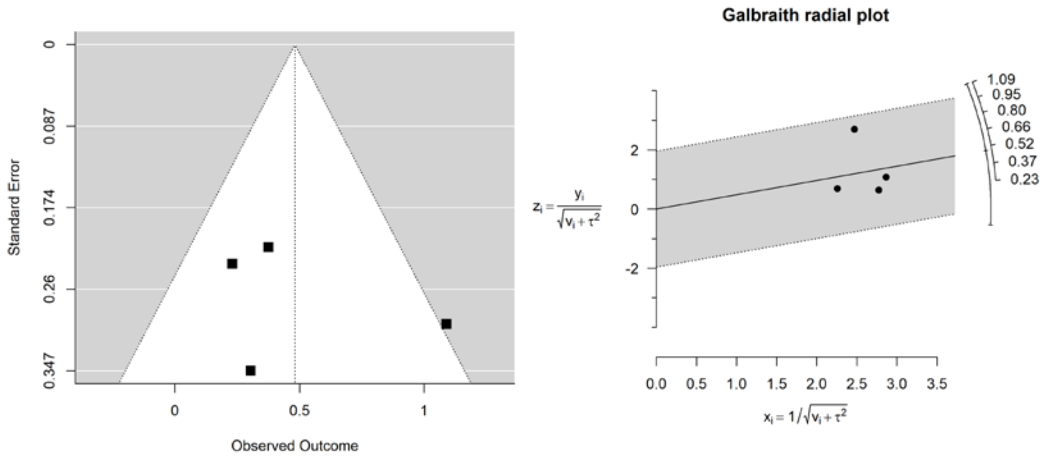


**Figure S4.** LANG.

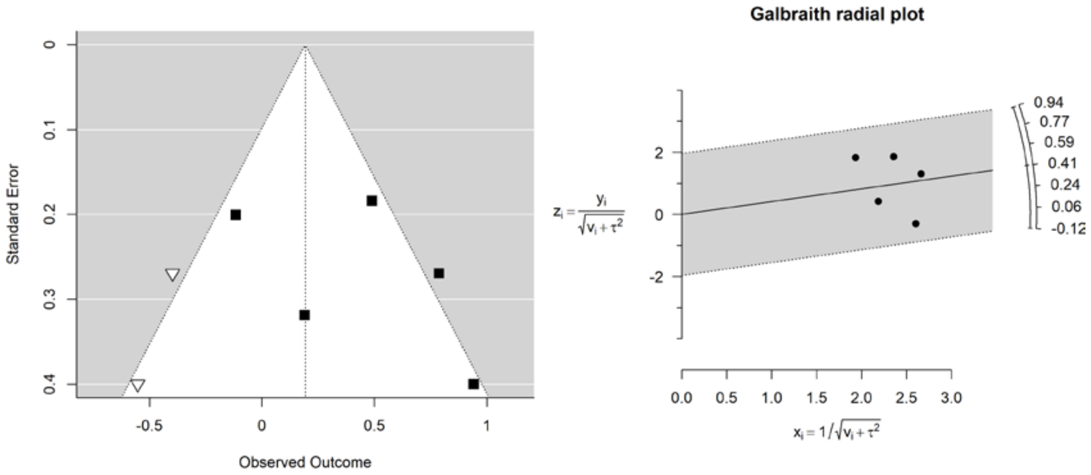


**Figure S5.** GLOBAL COGNITION.
